# A Role for Circular Non-Coding RNAs in the Pathogenesis of Sporadic Parathyroid Adenomas and the Impact of Gender-Specific Epigenetic Regulation

**DOI:** 10.3390/cells8010015

**Published:** 2018-12-30

**Authors:** Maria P. Yavropoulou, Christos Poulios, Nickos Michalopoulos, Ariadni Gatzou, Sofia Chrisafi, Stylianos Mantalovas, Theodosis Papavramidis, Emily Daskalaki, Electra Sofou, Kalliopi Kotsa, Isaak Kesisoglou, Pantelis Zebekakis, John G. Yovos

**Affiliations:** 1 1st Department of Propaedeutic Internal Medicine, National and Kapodistrian University of Athens, School of Medicine, Laikon General Hospital, Athens 11527, Greece; 2 Pathology Department, Faculty of Medicine, Aristotle University of Thessaloniki, Thessaloniki, 54124, Greece; dr.poulios@hotmail.com (C.P.); chrisafi@med.auth.gr (S.C.); emidask@gmail.com (E.D.); 3 3rd Department of Surgery, AHEPA Univ. Hospital, Aristotle University of Thessaloniki, Thessaloniki 54124, Greece; nickos.michalopoulos@gmail.com (N.M.); steliosmantalobas@yahoo.gr (S.M.); ikesis@hotmail.com (I.K.); 4 Laboratory of Clinical and Molecular Endocrinology, 1st Department of Internal Medicine, Aristotle University of Thessaloniki, Thessaloniki 54124, Greece; ariadnigatzou@gmail.com (A.G.); electrasofou@gmail.com (E.S.); kalmanthou@yahoo.gr (K.K.); pzebeka@med.auth.gr (P.Z.); giovos@med.auth.gr (J.G.Y.); 5 1st Department of Surgery, AHEPA Univ. Hospital, Aristotle University of Thessaloniki, Thessaloniki, 54124, Greece; papavramidis@hotmail.com

**Keywords:** parathyroid adenomas, pathogenesis, epigenetics, non-coding RNAs, circular RNAs

## Abstract

Epigenetic changes, including altered small non-coding RNAs, appear to be implicated in the pathogenesis of sporadic parathyroid adenomas (PAs). In this study, we investigated the circular RNAs (circRNAs) expression profile in sporadic PAs. Sixteen tissue samples of sporadic PAs, and four samples of normal parathyroid tissue (NPT) were investigated. Sample preparation and microarray hybridization were performed based on the Arraystar’s standard protocols, and circRNAs sequences were predicted by bioinformatics tools. We identified 35 circRNAs that were differentially expressed in sporadic PAs compared to NPT; 22 were upregulated, and 13 were downregulated, according to the pre-defined thresholds of fold-change **>** 2.0 and *p* **<** 0.05. In the subgroup analysis of PAs from male patients (n = 7) compared to PAs from female patients (n = 9), we also find a different expression profile. In particular, 19 circRNAs were significantly upregulated, and four circRNAs were significantly downregulated in male patients, compared to female counterparts. We show here for the first time a differential circRNA expression pattern in sporadic PAs compared to NPT, and a different expression profile in PA samples from male compared to female patients, suggesting an epigenetic role in the PA pathogenesis, and also an effect of gender in the epigenetic regulation of PAs.

## 1. Introduction

Primary hyperparathyroidism (PHP) is considered to be one of the most common endocrinopathies with a prevalence of 0.3% in the general population [1] showing a predilection for postmenopausal women [2]. PHP is characterized by hypercalcemia, hypercalciouria, hypophosphatemia, and high or inappropriately normal levels of serum PTH. The increased secretion of PTH from parathyroid cells in PHP is usually due to a benign monoclonal expansion of parathyroid cells, leading to parathyroid adenoma (PA) or hyperplasia, while malignancy rarely occurs and accounts for less than 1% of the cases [3]. 

The pathophysiology of parathyroid tumorigenesis involves inactivating germline mutations of suppressive oncogenes in the majority of cases with familial predisposition. In sporadic PAs, several genes, including multiple endocrine neoplasia type 1 (MEN1), cyclin D1/PRAD1, and cyclin-dependent kinase inhibitors (CDKI), such as CDKN1B/p27, have also been implicated in the pathogenesis [4].

Apart from alterations in MEN1, however, genetic abnormalities in other genes appear to occur very rarely, and thus the role of epigenetic changes in parathyroid tumorigenesis is under intensive investigation. 

Non-coding RNAs, including long–non-coding RNAs (lncRNAs), short micro-RNAs (miRNAs), and circular RNAs (circRNAs), control various levels of gene expression in physiology and disease, such as chromatin architecture/epigenetic memory, transcription, RNA splicing, editing, translation, and possibly tumorigenesis [5]. CircRNAs are a relatively newly-discovered type of non-coding RNAs that are formed from the covalent linkage of the 3’ and 5’ ends to form a closed loop [6]. As a result of this closed structure, circRNAs have been shown to be highly stable and largely resistant to RNA degradation pathways [7], which suggests that circRNAs may be more useful molecular biomarkers for human diseases than linear non-coding RNAs. 

Similar to miRNAs, several circRNAs have shown aberrant expression in human neoplastic diseases. As such, the expression of hsa_circ_0001141 and hsa_circ_001763 has been shown to be significantly silenced in squamous cell carcinoma of the esophagus and colorectal cancer tumors, while the expression of hsa_circ_002059 and hsa_circ_0001649 has been shown to be significantly down-regulated in gastric malignancies, and in hepatocellular carcinoma tumors, respectively [7]. Data are scarce, however, on the expression of circRNAs in parathyroid tissue. To address this issue, we profiled the circRNA tissue expression of sporadic PAs compared to normal parathyroid tissues, in order to gain a better insight on the role of epigenetic changes in the pathogenesis of sporadic PAs. 

## 2. Materials and Methods

### 2.1. Sample Selection

**Figure 1:**
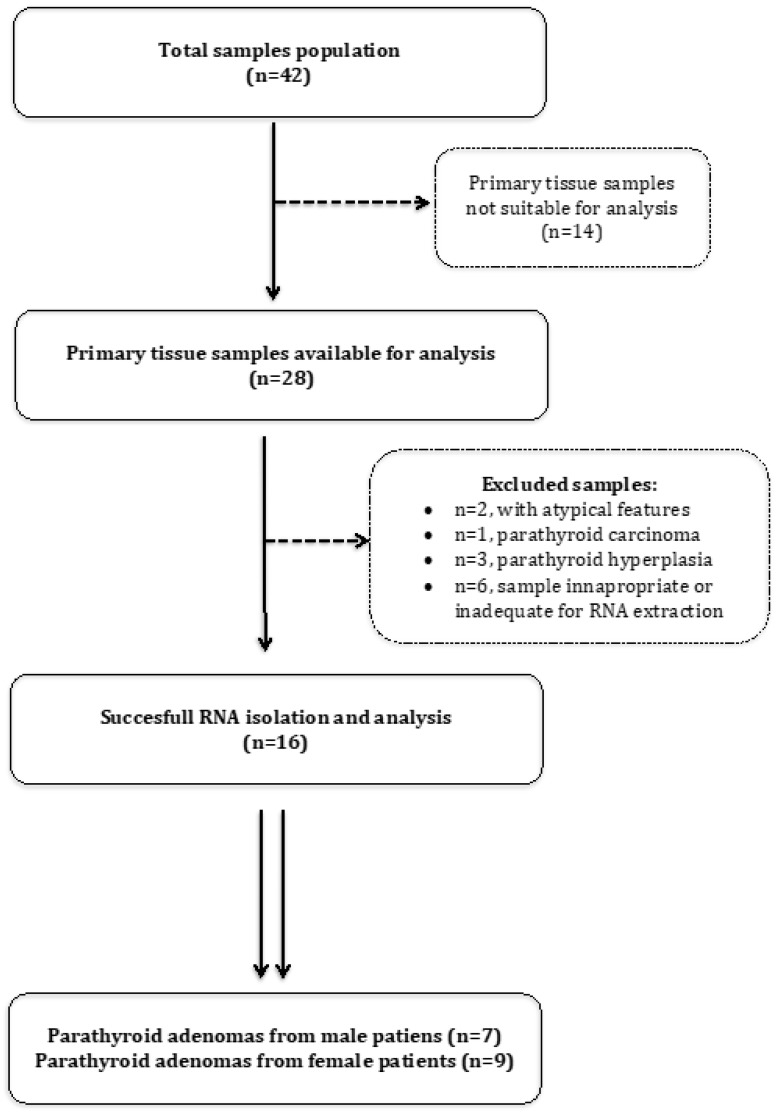
Flow chart detailing the selection of parathyroid adenomas (PA) tissue samples.

Parathyroid tissue samples, alongside with clinical and histopathological data, were collected from patients that underwent parathyroidectomy (PTX) from December 2014 to February 2017. Inclusion criteria were: single gland disease, occurring in adults with no family history of primary hyperparathyroidism nor a personal/family history suggestive of multiple endocrine neoplasia or a related syndrome, and no evidence of atypical or malignant features. Cases of parathyroid carcinomas according to Schantz and Castleman’s histologic criteria [8] and parathyroid hyperplasia were excluded from the final analysis. Sixteen patients with PAs that fulfilled the inclusion/exclusion criteria, and normal parathyroid tissue (NPT) samples from four patients, which were obtained at the time of total thyroidectomy for benign multinodular goiter, were finally included in the analysis (Figure 1). 

Tissue samples were not suitable for one of the following reasons: no tumor cells present in tumor samples (n = 7). Tissue samples were withdrawn for one of the following reasons: patients with atypical features (n = 3), analysis unsuccessful (n = 4).

We performed an immunohistochemical evaluation of the adenomatous polyposis coli (APC) gene, Ki67, cyclin D1, and parafibromin expression, in order to definitely establish a diagnosis of PA, and to exclude cases of atypical PA and/or parathyroid carcinoma [9,10,11]. The study was approved by the Scientific Committee of the affiliated AHEPA University Hospital.

### 2.2. Histopathological and Immunohistochemical Analysis

Tissue samples from all patients were collected, fixed in formaldehyde 10% and embedded in paraffin blocks (Formalin Fixed, Paraffin Embeded, FFPE). Sections (3 μm) from all the blocks were taken and stained with Hematoxylin–Eosin (H&E) for morphological evaluation and verification of adenoma or normal PTH, accordingly. In addition, the H&E slides were used for marking the epithelial rich areas for microdissection and tumor microarray (TMA) construction. TMAs constructed after the necessary slides for the below described microdissection were obtained. From each patient’s block, three cores were obtained, each 1 mm in diameter. The cores were taken from areas with high ratio of epithelial to stromal component. From the TMA blocks, one slide was taken and stained with H&E, in order to confirm that there was adequate tissue. Another four sections (3 μm each) from each TMA block was taken and stained for Ki67 (MIB1 clone, DAKO, Glostrup, Denmark), CyclinD1 (EP12 clone, DAKO), APC (C-20 clone, Santa Cruz Biotechnology Inc., Dallax, TX, USA), and parafibromin (2H1 clone, Santa Cruz Biotechnology Inc.). All immunohistochemical stains were performed by using Bond Max and Bond III autostainers (Leica Microsystems, Wezlar, Germany). Parafibromin was evaluated as “positive” for any percentage of nuclear positivity, and “negative” for no nuclear positivity. For APC, cytoplasmic positivity was evaluated as a percentage of positive cells. For CyclinD1 and Ki67, nuclear positivity was evaluated as a percentage of positive cells. Each stain was evaluated in three cores, and the average value was taken into account for statistical analysis. 

### 2.3. RNA Extraction from Paraffin-Embedded Tissue

Manual microdissection was performed from each FFPE block to enrich for epithelial tissue before total RNA extraction. In brief, one H&E slide and up to five unstained slides (10 μm thickness) were generated from each FFPE block. The target lesion was marked on the H&E slide for each case, which was then used to guide the removal of non-target tissues (e.g., normal parathyroid tissue) from unstained slides. After an overnight deparaffinization, the tissue area of interest was scraped off the slide into a microcentrifuge tube to facilitate total RNA extraction. RNA extraction was performed by miRNeasy FFPE kit (Qiagen GmbH., Hilden, Germany), according to manufacturer’s instructions. The kit is optimized to isolate RNA molecules that are longer than 18 nucleotides from FFPE tissue samples, and to reverse as much formaldehyde modification as possible without further RNA degradation. 

The concentrations of the RNA samples were determined by OD_260_ by using a NanoDrop ND-1000 instrument. The integrity of RNA was assessed by electrophoresis on a denaturing agarose gel.

### 2.4. Sample Labeling and Microarray Hybridization

The sample preparation and microarray hybridization were performed based on the Arraystar’s standard protocols (Arraystar Inc., Rockville, MD, USA). All samples were used for microarray analysis.

Briefly, total RNA from each sample was digested with Rnase R (RNase R; Epicenter, Madison, WI, USA) to remove linear RNAs, and to enrich circular RNAs. Then, the enriched circular RNAs were amplified and transcribed into fluorescent complement RNA utilizing a random priming method (Arraystar Super RNA Labeling Kit; Arraystar, Rockville, MD, USA). The fluorescently-labeled cRNAs were quantified with a NanoDrop ND-1000 spectrophotometer (NanoDrop) and hybridized onto the Arraystar Human circRNA Array V2 (8x15K, Arraystar) (Figure S1). After having washed the slides, the arrays were scanned by the Agilent Scanner G2505C. Agilent Feature Extraction software (version 11.0.1.1) was used to analyze acquired array images. CircRNA sequences were predicted by bioinformatic methods [12].

### 2.5. Quantitative Real Time Reverse Transcription Polymerase Chain Reaction (qRT-PCR)

Following RNA extraction from parathyroid adenomas and normal parathyroid tissue cDNA was synthesized with a reverse transcriptase according to the kit’s instructions (miScript II RT Kit, Qiagen). In brief, we used 1 ug RNA, polyadenylated by poly(A) polymerase and converted it into cDNA by reverse transcriptase with oligo-dT priming using the miScript HiFlex Buffer. The 18S and 28S ribosomal RNAs were used as internal standards. Cycling was performed under standardized conditions with 2× QuantiTect® SYBR Green PCR Master Mix on the QIAGEN Rotor-Gene Q (Corbett Rotor-Gene 6000, Hilden, Germany) real-time PCR cycler. Relative circRNA expression was calculated with the 2^−ΔΔCt^ method.

### 2.6. Prediction of circRNA–miRNA Interactions

Circular RNAs have been shown to affect miRNA-mediated regulation of gene expression through miRNA sponging [13]. Therefore, the target prediction software, based on Circbase 2.0, TargetScan and Circular RNA Interactome (NIH), was applied to predict putative circRNA/miRNA interactions for the differentially expressed circRNAs in PAs compared to NPT, identified from the microarray hybridization. The Circ-Interactome is a computational tool that enables the prediction and mapping of binding sites for RNA binding proteins (RBP) and miRNAs on reported circRNAs. This tool searches mammalian circRNAs’ names and the genomic position and the best-matching transcripts of the circRNA. Then, it retrieves genomic and mature circRNA sequences and searches RBPs binding to a circRNA, and to sequences upstream/downstream of the circRNA. Finally, it identifies RBPs binding to the circRNA junctions and miRNAs targeting a circRNA [14].

#### Prediction of circRNA–miRNA–Target Gene Associations

Several online bioinformatics platforms such as TargetScan, CircBase, MirGator, MirBase, CircNet, and NIH Circ interactome were applied in conjunction in order to predict circRNA- miRNA-target gene associations. 

### 2.7. Statistical Analysis

Quantile normalization and subsequent data processing were performed, using the R software limma package. Differentially expressed circRNAs with statistical significance between two groups were identified through Volcano Plot filtering. Differentially expressed circRNAs between two samples were identified through Fold Change filtering. Hierarchical Clustering was performed to show the distinguishable circRNAs expression pattern among samples. When comparing the two groups (PAs versus control), for profile differences, we computed the “fold change’’ between the groups for each circRNA. circRNAs having more than 2-fold changes and *p*-values < 0.05 were selected as being significantly differentially expressed.

## 3. Results

Mean age of the included patients was 60 ± 8 years, including nine postmenopausal females and seven males. The control group included samples of NPT from two males and two females mean age 65.7 ± 3.7 years. The demographic and clinical characteristics of the included patients are depicted in Table 1. 

As expected, patients with PHP had significantly higher levels of serum calcium and PTH levels compared to controls (sCa: 2.7 ± 0.2 mmol/L vs. 2.28 ± 0.09 mmol/L, *p* < 0.001, and sPTH: 12.68 ± 3.5 pmol/L vs. 4.42 ± 0.57 pmol/L, *p* < 0.001, respectively). None of the patients had significant level of hypophosphatemia, although serum phosphate was in the low normal range. After parathyroidectomy, there was a complete biochemical remission of primary hyperparathyroidism in all patients with serum PTH, calcium, and PO_4_ reaching normal values within one to two months post-surgery.

### 3.1. Immunohistochemical Findings

**Figure 2:**
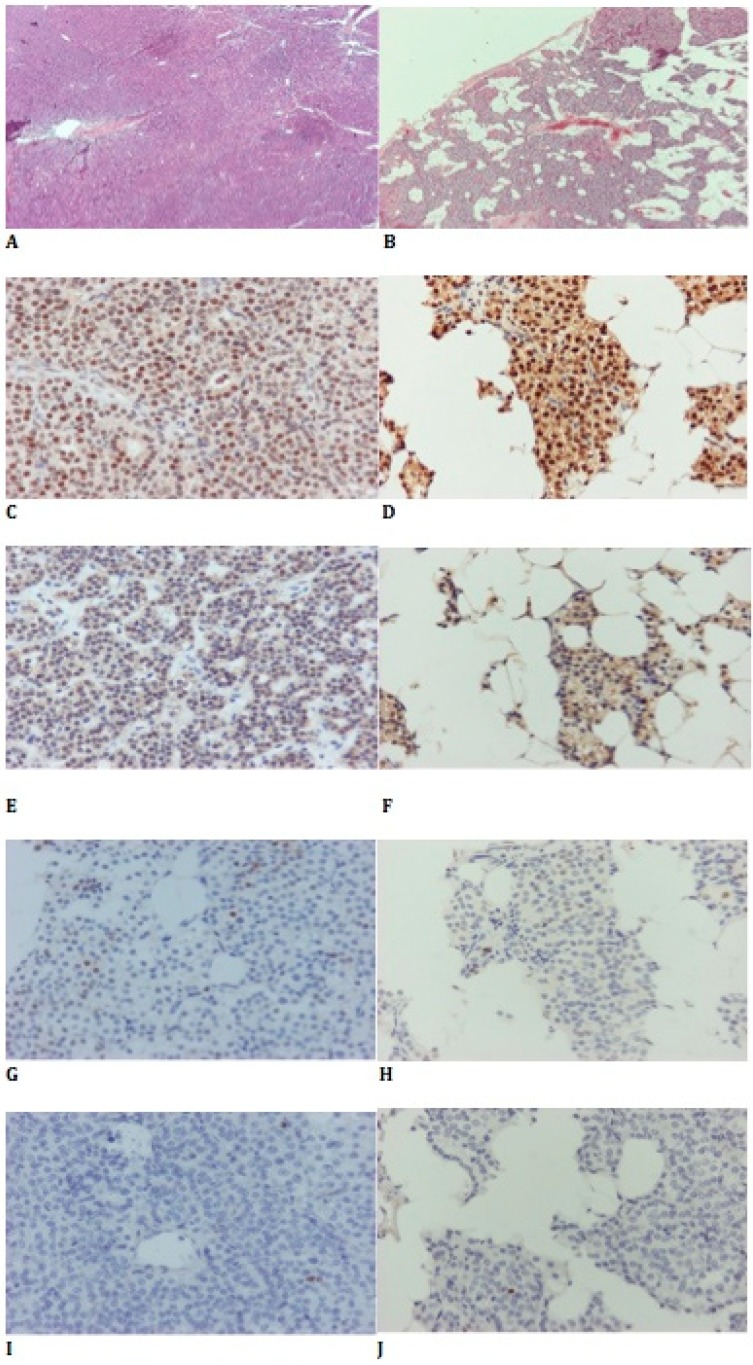
Immunohistochemical staining of sporadic PA and control samples. Representative images of H&E-stained 40× (**A**) PA samples, (**B**) control samples, parafibromin-stained 400×, (**C**) PA samples, (**D**) control samples, APC-stained 400× (**E**) PA samples, (**F**) control samples, Cyclin D1-stained 400×, (**G**) PA samples, (**H**) control samples, ki67-stained 400× (**I**) PA samples, (J) control samples. PA, parathyroid adenomas; H&E, Hematoxylin-Eosin; APC, adenomatous polyposis coli.

Representative images of H&E-stained parathyroid tumor specimens are provided in Figure 2. All tested samples, both PAs and NPT were positive for parafibromin (Figure 2, Table S1). Loss of APC expression was found in one case of PA, while all other cases were positive. Ki67 expression was low in all cases of PAs, reaching up to 1%, and negative in all NPT samples. Expression of cyclinD1 was significantly higher in PAs compared to NPT, as previously described [15,16]. 

Immunohistochemical findings confirmed the diagnosis of PAs.

### 3.2. Identification of Differential circRNA Profiles

**Figure 3:**
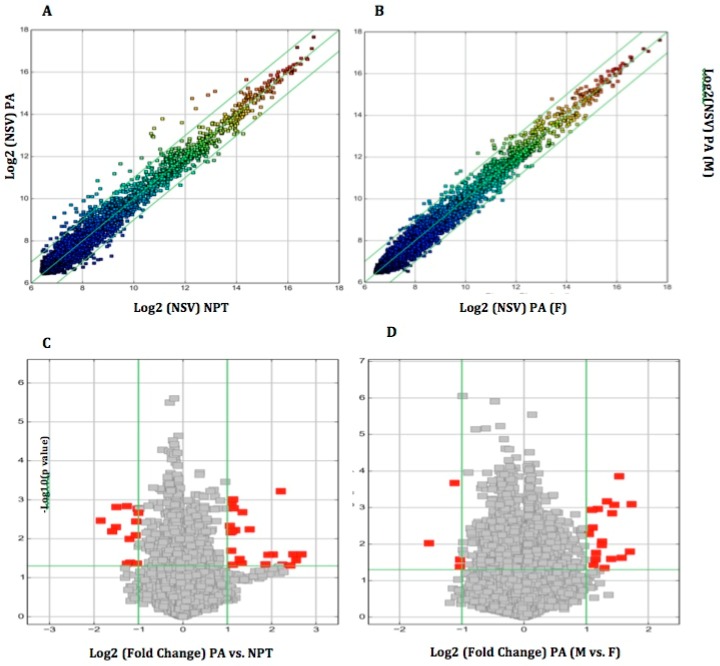
Scatter plots and volcano plots to identify differentially-expressed circular RNAs (circRNAs). Scatter plots used to identify differentially-expressed circRNAs in (**A**) PAs vs. NPT and (**B**) PAs from F patients vs. PAs from M patients. The y-axis represents the mean normalized circRNA signal values for each comparator group (log2-scaled). The green fold-change lines represent 2.0× fold-changes, so the circRNAs lying above and below these green lines displayed greater than a 2.0-fold upregulation or downregulation. Volcano plots used to identify differentially-expressed circRNAs in (**C**) PAs vs. NPT and (**D**) PAs from F patients vs. PAs from M patients. The x-axis represents fold-change values (log2-scaled), while the y-axis represents *p*-values (−log10-scaled). The green vertical lines correspond to 2.0× upregulation and downregulation, respectively, while the green horizontal line corresponds to a *p*-value of 0.05. On this basis, the red rectangles represent the differentially-expressed circRNAs of statistical significance. NSV, normalized signal value; PAs, parathyroid adenomas; NPT, normal parathyroid tissue; F, female; M, male.

The Arraystar Human circRNA Microarray was used to sample labeling and microarray hybridization of all the finally selected parathyroid-tissue samples (Figure 3). 

We identified 35 circRNAs that were differentially expressed in sporadic PAs compared to normal parathyroid tissue; 22 were upregulated, and 13 were downregulated (Table 2), according to the pre-defined thresholds of fold-change > 2.0 and *p* < 0.05. Among upregulated circRNAs; hsa_cirRNA_051778, hsa_cirRNA_402533, hsa_cirRNA_406174, and hsa_cirRNA_0008267 showed the highest fold-change (>5-fold), whereas hsa_circRNA_032603 and hsa_circRNA_058097 showed the highest fold-change (>3-fold) among the downregulated circRNAs.

### 3.3. qRT-PCR Validation of Differential circRNAs

**Figure 4:**
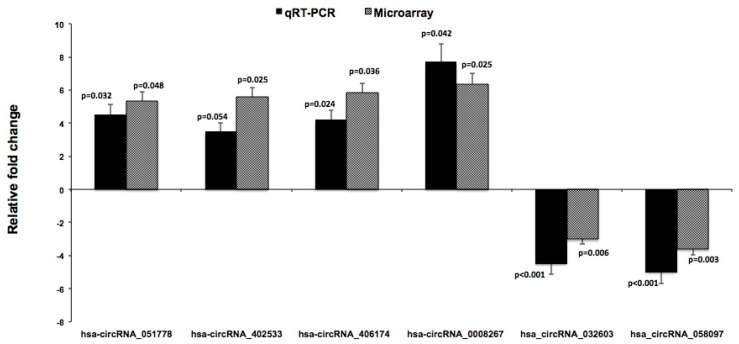
qRT-PCR validation of differentially expressed circRNAs in PAs. qRT-PCR was used to validate the dysregulated expression of the four significantly upregulated and the two significantly downregulated circRNAs that showed the highest fold-expression in PAs (n = 16) vs. normal parathyroid tissue (n = 4) in the microarray analysis. The same samples used for microarray analysis were also used for qPCR validation. The y-axis represents the relative fold change compared to controls. Data are displayed as means ± SEMs. *p* values for Microarray and qRT-PCR analysis are indicated in the figure.

The four significantly upregulated circRNAs and the two significantly downregulated circRNAs with the highest fold change were selected for qRT-PCR validation in the same samples that were used for the microarray analysis. The qRT-PCR results revealed that all six circRNAs but one (namely hsa_circ_RNA_402533) were significantly differentiated between PAs vs. normal parathyroid tissue, with fold-changes mirroring those derived from the microarray data (Figure 4). 

### 3.4. Construction of circRNA-miRNA Network

As circRNAs interact with miRNAs via miRNA response elements (MREs), we searched for putative MREs through Arraystar’s circRNA target prediction software, and CircBase, CircNet, and NIH Circ interactome. The predicted miRNAs for the significantly-upregulated and downregulated circRNAs are listed in Table 2, with hsa-miR_1248 being the most frequently targeted miRNA by 5 up-regulated circRNAs (hsa_circRNA_404337, hsa_circRNA_100965, hsa_circRNA_101283, hsa_circ-RNA_007273, hsa_circ-RNA_406174) and one downregulated (hsa_circRNA_021732). Other less frequently targeted miRNAs, include hsa_miR-139-5p, targeted by four upregulated circRNAs (hsa_circRNA_102904, hsa_circRNA_102903, hsa_circRNA_101283, hsa_circRNA_002617), hsa_miR_1264, targeted by three upregulated circular RNAs (hsa -circRNA_404337, hsa_circRNA_100965, hsa_circRNA_002127) and hsa_miR-486-3p targeted by three down-regulated circRNAs (hsa_circRNA_404643, hsa_cirRNA_105038, hsa_cirRNA_032603).

We also searched for the total miRNA binding site (miRBS) that has been reported so far for the three most upregulated and the two most down-regulated circRNAs. We identified 46 miRBS for hsa_circRNA_008627; 41 for hsa_circRNA_058097; 18 for hsa_circRNA_406174; seven miRBS for hsa_circRNA_032603; one for hsa_circRNA_402533.

### 3.5. circRNA–Genes Interactions

To further explore the underlying molecular mechanism(s) of the differentially expressed circRNAs in sporadic PAs, we used the bioinformatics platforms CircBase, and NIH Circ-Interactome to predict circRNA–genes interactions. The predicted genes associated with the differentially expressed circ-RNAs are depicted in Table 2. Two circRNAs, hsa_circRNA_404337 and hsa_circRNA_051799, interact with genes that have been shown to associate with molecular pathways that are involved in the pathogenesis of sporadic PAs, such as CDKN1B and CDK1, respectively [17,18].

Most of the genes identified are protein-coding, and associated with several cellular processes, such as organization and re-arrangement of the cytoskeleton, cell proliferation, cell metabolism, and transcription (Table S2). 

### 3.6. Parathyroid Adenomas from Male vs. Female Patients

In order to test whether the gender of the patient has an impact in the expression profile of circRNAs in PAs, since the vast majority of PHP due to PAs appear in postmenopausal women, we performed a sub-group analysis of PAs from male patients compared to PAs from female patients. We found a different expression profile between male and female patients. In particular 19 circRNAs were significantly upregulated and four circRNAs were significantly downregulated in male patients compared to female counterparts (Table 3). It is worth noting that the identified differentially expressed circRNAs in the subgroup analysis between male and female patients were different from the ones that we have identified in our initial analysis between PA samples and controls, and there was no overlap in circRNAs expression profiles between the two analyses.

The predicted miRNAs for the significantly upregulated and downregulated circRNAs are also listed in Table 3, with hsa_miR_1184 being the most frequently targeted miRNA by 4 up-regulated circRNAs (hsa_circRNA_008636, hsa_circRNA_000763, hsa_circRNA_092388, hsa_cirRNA_044837) and one down-regulated circRNA (hsa_circRNA_100332). The predicted genes associated with the differentially expressed circRNAs in this analysis are also depicted in Table 3. The majority of these genes are protein coding (Supplemental Table S3) and involves the intracellular ubiquitin proteasome system, nucleic acid and RNA binding, protein heterodimerization and homodimerization activity, vesicle trafficking and alternative splicing of pre-mRNAs. None of the implicated genes, however, is located in the X chromosome (Table 3), and thus we cannot address whether incomplete inactivation of the X chromosome is a possible mechanism, contributing to the altered expression of some circRNAs in males vs. females. 

In order to further evaluate our results in the differential expression of sporadic PAs in male vs. female patients, we also conducted a subgroup analysis between samples from males and females in the control group, to test whether the altered expression of circRNAs profile is disease-independent. We did not find any significant difference in this analysis, suggesting that the differences in circRNA expression between male and female patients may be attributed to PA, but the numbers were small. 

## 4. Discussion

In this study, we investigated the circRNA expression profile in sporadic PAs, and we tested the effect of gender. 

Thirty-five circRNAs, 22 up-regulated, and 13 down-regulated were identified by microarray analysis to be differentially expressed in sporadic PAs compared to NPT. Applying a set of bioinformatic tools, we constructed a network of circRNA–miRNA and genes interactions, identifying two of the differentially expressed circRNAs, hsa_circRNA_404337 and hsa_circRNA_051799, that interact with genes known to associate with molecular pathways involved in the pathogenesis of sporadic PAs, such as CDKN1B and CDK1 [4,19], respectively. In the sub-analysis within the cohort of sporadic PAs, we also report a differential circRNA expression profile in sporadic PAs from male patients compared to PAs from female counterparts with 19 circRNA being significantly upregulated and four being significantly downregulated. Primary hyperparathyroidism is most frequently present in postmenopausal women, and although the involvement of estrogen has been suggested, and parathyroid tumors are found to express ERβ1 and ERβ2 [20], the exact underlying pathophysiology remains obscure. The differences in circRNA expression profile between male and female patients, but not between male and female controls, point to a potential gender effect on the PA tumorigenesis through epigenetic regulation. 

In the past decades an ever-growing list of diverse non-coding RNAs with functional capacity have been identified in eukaryotic cells [21]. CircRNAs are formed by alternative splicing of pre-mRNA, in a process known as ‘backsplicing’, where an upstream splice acceptor is joined to a downstream splice donor [22]. They are expressed in a variety of eukaryotic organisms, demonstrate conservation across mammals, they are abundant, expressed in a regulated manner independent of their cognate linear isoform and in a cell type-specific manner.

Dysregulation of circRNAs profile has been identified in various types of cancers, such as papillary thyroid carcinoma [23], gastric cancer [24], hepatocellular cancer [25,26,27], colorectal cancer [28,29,30], bladder cancer [31], and lung cancer [32], as well as in non-cancer conditions including cardiovascular disease [33], Alzheimer’s disease [34], and diabetes [35,36]. The implication of tissue-specific circRNAs, however, in disease pathogenesis, progression, and therapy resistance remains largely unknown, and is currently under intensive investigation [37]. 

**Figure 5:**
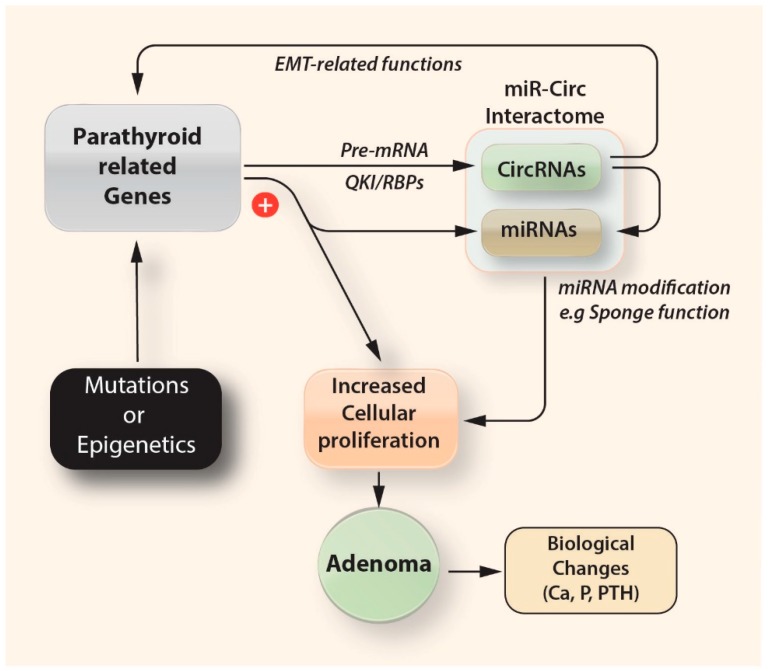
Stochastic approach of the potential interactions between genes and non-coding RNAs, in the pathogenesis of parathyroid adenomas. Some circRNAs play critical roles in fine-tuning the level of miRNA-mediated regulation of gene expression by sequestering the miRNAs. miR: microRNAs; circ: circular RNAs; EMT: epithelial–mesenchymal transition; RBPs: RNA-binding proteins, QKI: Quaking homolog, KH domain RNA binding (RNA binding protein).

In cancer, circRNAs appear to have a role in cancer development and progression, by interacting with microRNAs. CircRNAs negatively regulate the inhibitory effects of miRNAs upon their target linear mRNAs by directly binding to miRNAs via MREs, thereby essentially functioning as ‘miRNA sponges’ within the cellular RNA network. Another potential function of circRNAs could be to compete with the splicing of an mRNA [38], while some circRNAs have also been implicated in transcriptional or post-transcriptional gene regulation of their host genes [39] (Figure 5). Compared to mRNAs, circRNAs are more stable, due to their natural resistance to exonucleases, with half-lives greater than 24–48 h, which is significantly longer than the half-life of an average mRNA (8-9 h in human cells) [40,41]; this is why they are considered as being much more promising in understanding the pathophysiology of certain diseases and the development of cancer.

The emerging role of the epigenetic component in tumorigenesis has just now started to be identified in parathyroid neoplasia [6,19].

MiRNA profiles have been investigated in parathyroid tissue before, demonstrating dysregulated miRNA expression profile in parathyroid carcinomas [6,42], and showing a global down-regulation (approximately 80%) of miRNAs compared to normal parathyroid tissue. Other studies have also identified significantly up-regulated miRNAs, such as miR-222 [6] miR-503 [6], and miR-517c [43] in parathyroid carcinomas. 

The mature form of miRNA_24-1 was studied in the parathyroid hyperplasia of MEN1. It has been shown that miRNA_24-1 is uniquely expressed in MEN1 parathyroid adenoma tissues that conserve the wild-type allele, while it is downregulated in the MEN1 parathyroid adenoma tissues with the 11q13 LOH, as well as in sporadic parathyroid adenomas [44]. 

We also search for miRNA response elements (MREs) on the 35 differentially expressed circRNAs, which were then used to identify putative miRNAs that were based on their seed-sequence complementarity [13]. Hsa_miR_1248 was the most frequently encountered miRNA, and although little is known about the potential targets and pathways regulated by this miRNA it has been linked to the pathogenesis of breast [45] and non-small cell lung carcinoma [46], autism spectrum disorders [47], asthma [48], and in the aging process [49]. Genome-wide microarray and in vitro studies [48,49], have shown that miRNA-1248 regulates the expression of genes that play a role in interleukin-related inflammatory pathway, as well as, DNA repair pathways.

None of the miRNAs that were previously reported in parathyroid carcinomas, however, was identified in our analysis through interaction with the reported dysregulated circRNAs. 

Apart from the dysregulated expression of non-coding RNAs, the hypermethylation of APC, the gene responsible for familial adenomatous polyposis, occurs frequently in parathyroid adenomas although aberrant APC expression has not been demonstrated [50,51]. In addition, genes that are frequently subject to epigenetic inactivation in human cancers, such as Ras association domain-containing protein 1 (RASSF1A) [52] and hypermethylated in cancer 1 protein (HIC1) [53], are also frequently hypermethylated in parathyroid adenomas, but it is unclear which of these aberrantly methylated genes may be important to the pathogenesis of parathyroid tumors. 

Our study has several limitations; (i) the sample size was relatively small, (ii) our analysis was limited to parathyroid tissue, and thus results from other biosamples such as serum or plasma before and after parathyroidectomy that could strengthen our results are missing and (iii) we were not able to perform DNA analysis and investigation of somatic mutations in our samples, mainly due to inadequate material in the total of PA samples used. 

Despite these limitations, however, this is the first study investigating the circRNA expression profile in sporadic PAs. We identified 35 differentially expressed circRNAs in PA samples compared to normal parathyroid tissue, suggesting a role of circRNAs in the pathogenesis of these benign tumors. In addition, we reported significant differences in the circRNA expression profile between male and female patients underlying the fact that epigenetics may be involved in the gender specific differences reported in PAs. Furthermore, our results from the bioinformatic analysis reveal a circRNA–miRNA network that could play a key role in the triggering of the tumorigenetic process in the parathyroid cells. As circRNAs regulate the epigenome in multiple ways (Figure 5), future functional studies that can pave the way between ‘circnome’ and PA pathogenesis would significantly increase our knowledge for the molecular pathways in involved.

CircRNA network appears to be cell and tissue specific [37], stable, and with a significant regulatory role in pathogenesis of diseases, providing new strategic approaches to tumorigenesis, and emerge as promising diagnostic and prognostic biomarkers, and as therapeutic targets.

## Figures and Tables

**Table 1:** Demographic and biochemical profile of included patients and healthy controls.

Patient Code	Age	Baseline Values *				Post-PTX Values #	
		s Ca	sPO_4_	iPTH	s Ca	sPO_4_	iPTH
		NR: 2.10–2.50 mmol/L	NR: 1.0–1.5 mmol/L	NR: 1.4–6.8 pmol/L	NR: 2.1–2.5 mmol/L	NR: 1.0–1.5 mmol/L	NR: 1.4–6.8 pmol/L
Di_M_27	54	3.11	1.2	18.4	2.63	1.9	0.6
Di_M_28	64	2.72	1.4	7.6	2.65	2	1.2
Di_M_32	70	2.71	0.9	18.7	2.9	1.5	1.5
Di_M_35	48	2.85	1.23	11.6	2.3	1.2	0.6
Di_M_36	48	3.08	1.09	12.9	2.31	1.43	1.7
Di_M_37	78	2.63	1.1	18.5	1.88	1.67	1
Di_M_38	56	2.68	1.4	11.5	2.23	1.8	2.2
Di_F_9	65	3.27	1.5	12.7	2.63	1.39	0.6
Di_F_10	65	2.75	1.4	12.1	2.31	1.44	0.6
Di_F_11	58	2.74	0.9	16.2	2.45	1.23	4.2
Di_F_12	58	2.69	1.2	10.3	2.2	1.3	3.5
Di_F_14	67	2.68	1.12	9.5	1.93	1.3	0.6
Di_F_15	67	2.48	1.16	13.4	1.9	1.6	3.4
Di_F_20	58	2.64	1.22	10.8	2.34	1.48	2.8
Di_F_21	57	2.75	1.1	8.6	2.14	1.65	1.4
Di_F_34	52	2.63	1.09	10.2	2.09	1.3	4.3
Con_1M	65	2.23	1.4	4.3	/	/	/
Con_2M	71	2.41	1.45	3.8	/	/	/
Con_3F	65	2.19	1.32	4.4	/	/	/
Con_4F	62	2.29	1.25	5.2	/	/	/

* Baseline values: were measured within two months prior to parathyroidectomy. # PostPTX values: were measured 1–2 months after parathyroidectomy. M, male; F, female; sCa, serum calcium; sPO_4_, serum phosphate; iPTH, intact parathyroid hormone; NR, normal range; PTX, parathyroidectomy.

**Table 2:** Circular RNAs with significantly altered expression in parathyroid adenomas compared to normal parathyroid.

CircRNA	CircRNA ID ‡	Fold Change	*p* Value	FDR	Chr.Gene/Symbol	hsa-miRNA targets ‡‡
**Up-regulated CircRNAs**						
hsa-CircRNA_102904	hsa_Circ_0008459	4.6	0.001	0.126	KANSL1L/C2orf67(Chr.2)	139-5p, 520f, 144, 1206, 146b-3p
hsa-CircRNA_404337	hsa_Circ_0140094	2.14	0.001	0.148	RPS6KA3 (Chr. X)	1248, 1261,1264, 331-3p, 623, 644
hsa-CircRNA_043614	hsa_Circ_0043614	2.18	0.046	0.482	KRT14 (chr.17)	1184, 1205, 338-3p, 361-3p, 1224-3p, 766
hsa-CircRNA_102903	hsa_Circ_0002617	2.26	0.006	0.23	KANSL1L/C2orf67 (Chr.2)	127-5p, 1290, 139-5p, 149, 409-3p
hsa-CircRNA_100965	hsa_Circ_0024501	2.21	0.002	0.153	DDX6 (Chr.11)	1248, 1251, 1264, 577, 607, 660
hsa-CircRNA_001379	hsa_Circ_0000516	2.11	0.007	0.234	RPPH1 (Chr.14)	1296
hsa-CircRNA_402533 **	-	5.58	0.025	0.387	OVOL2 (Chr.20)	500b-3p
hsa-CircRNA_003949	hsa_Circ_0003949	2.83	0.006	0.23	PTN (Chr.7)	1256, 145, 326, 330-5p, 620
hsa-CircRNA_102116	hsa_Circ_0003258	3.79	0.026	0.392	ZNF652 (Chr.17)	502-5p, 203, 1278, 653, 766
hsa-CircRNA_404504	hsa_Circ_0141586	2.43	0.033	0.424	STIL (Chr.1)	-
hsa-CircRNA_051778	hsa_Circ_0051778	5.34	0.048	0.487	BCAT2 (Chr.19)	-
hsa-CircRNA_400537	hsa_Circ_0093450	2.08	0.005	0.223	MASTL (Chr.10)	1236, 1279, 1825,223, 346, 520
hsa-CircRNA_101283	hsa_Circ_0030586	2.51	0.043	0.465	ABCC4 (Chr.13)	1238, 1248, 217, 651, 767-5p, 139-5p
hsa-CircRNA_007273	hsa_Circ_0007273	2.19	0.001	0.146	RAB11FIP5 (chr.2)	1243, 1248, 1288, 361-3p, 876-3p
hsa-CircRNA_401782	hsa_Circ_0043138	4.71	0.046	0.479	TAF15 (Chr.17)	1257, 556-5p, 758, 1282, 767-3p
hsa-CircRNA_002122	hsa_Circ_0002122	4.07	0.025	0.391	CTCF (Chr.16)	1264, 136, 561, 644, 874
hsa-CircRNA_002617	hsa_Circ_0002617	2.13	0.02	0.36	KANSL1L/C2orf67 (Chr.2)	127-5p, 1290, 139-5p, 149, 409,
hsa-CircRNA_406174 ***	hsa_Circ_0005622	5.84	0.036	0.436	PITPNB (Chr.22)	604, 589, 1248, 593, 1248
hsa-CircRNA_104269	hsa_Circ_0078768	2.16	0.006	0.234	FAM120B (Chr.6)	1292, 661,1182, 1272, 1322
hsa-CircRNA_004286	hsa_Circ_0004286	2.53	0.002	0.162	SUSD1 (Chr.9)	1299, 182,31, 330-3p, 384
hsa-CircRNA_406165	-	3.62	0.045	0.477	COL6A2 (Chr.21)	-
hsa-CircRNA_008267	hsa_Circ_0008267	6.36	0.025	0.387	LINC00969/SDHAP2(Chr.3)	145, 1304, 198,498, 581
**Down-regulated CircRNAs**						
hsa-CircRNA_404643	hsa_Circ_0111746	2.31	0.01	0.272	PIK3C2B (Chr.1)	1224-3p, 1272, 1280, 486-3p, 586
hsa-CircRNA_105038	hsa_Circ_0105038	2.84	0.005	0.229	FLNA (Chr. X)	1225-3p, 1247, 149, 486-3p, 661
hsa-CircRNA_101474	hsa_Circ_0101474	2.03	0.002	0.153	THBS1 (Chr.15)	1322, 507, 557, 578, 615-5p, 663b
hsa-CircRNA_405040	hsa_Circ_0099504	2.4	0.001	0.153	PLXNC1 Chr.12	570, 1229, 513a-5p, 873, 1322
hsa-CircRNA_101051	hsa_Circ_0026143	2.27	0.041	0.458	TROAP Chr.12	637, 887, 1286, 661, 1247
hsa-CircRNA_032603	hsa_Circ_0032603	3.01	0.006	0.234	LTBP2 Chr.14	338-3p, 766, 940, 657, 486-3p
hsa-CircRNA_058097	hsa_Circ_0058097	3.61	0.003	0.198	FN1 Chr.2	876-3p, 1231, 1238, 515-5p, 558
hsa-CircRNA_074530	hsa_Circ_0074530	2.08	0.004	0.2	CD74 Chr.5	136, 145, 519e, 515-3p,767-3p
hsa-CircRNA_092437	hsa_Circ_0000741	2.4	0.044	0.472	POLR2A Chr.17	574-5p, 637, 604, 7, 490-5p
hsa-CircRNA_021732	hsa_Circ_0111746	2.82	0.002	0.153	CD44 Chr.11	1248, 1236, 145, 370, 636
hsa-CircRNA_101017	hsa_circ_0025506	2.01	0.002	0.162	GPRC5A Chr.12	1252, 296-5p,583, 873, 940
hsa-CircRNA_001766	hsa_circ_0001766	2.09	0.008	0.247	PDIA4 Chr.7	182, 1203, 136, 182, 766
hsa-CircRNA_102368	hsa_circ_0047663	2.04	0.043	0.467	RPL17 Chr.18	1204, 1208, 1228, 586

‡: The experimentally assayed circRNAs were cross-checked for the genomic position and the best transcript in circBase (http://circbase.org). ‡‡: The circRNA -corresponding hsa-miRNA targets with the best context+score percentile (NIH Circular RNA interactome). All identified circularRNAs are exonic arising from the exons of the linear transcript except for: **: Antisense, where their gene locus overlaps with the linear RNA, but is transcribed from the opposite strand. ***: Sense overlapping, transcribed from same gene locus as the linear transcript, but not classified into "exonic" and "intronic". has: *Homo sapiens*; Circ: circular; chr: chromosome.

**Table 3:** Circular RNA expression profile in parathyroid adenomas from male vs. female patients.

CircRNA	CircRNA ID‡	Fold Change	*p* Value	FDR	Gene Symbol/	hsa-miRNA targets‡‡
					Chr.	
**Up-regulated CircRNAs**						
hsa-CircRNA_401977	hsa_circ_0108703	2.67	0.001	0.09	NEDD4L	1231,1298, 149, 330-3p, 421, 561
					Chr.18	
hsa-CircRNA_103224	hsa_circ_0063329	2.52	0.001	0.07	DDX17	191, 571, 605, 622, 640
					Chr.22	
hsa-CircRNA_102850	hsa_circ_0001081	2.15	0.003	0.12	DCAF17	558,624,653,1200,1224-3p
					Ch.2	
hsa-CircRNA_008636 ***	hsa_circ_0008636	2.23	0.017	0.21	AAGAB	1256, 330-3p, 526b, 1182,1184
					Chr.15	
hsa-CircRNA_000763 **	hsa_circ_0007630	2.64	0.025	0.24	MBNL1	1184, 1263, 1305, 192, 215
					Chr.3	
hsa-CircRNA_026646	hsa_circ_0026646	2.28	0.001	0.08	PCBP2	1225-3p, 184, 1225-5p, 1233, 1245
					Chr.12	
hsa-CircRNA_406841 **	-	2.06	0.005	0.13	MIR5695	-
					Chr.19	
hsa-CircRNA_092388 ***	hsa_circ_0000315	3.26	0.016	0.2	INCENP	767-3p, 942, 1184, 578, 622
					Chr.11	
hsa-CircRNA_089762	hsa_circ_0089762	2.89	<0.001	0.04	JA760602	384, 554, 1249, 1283, 21
hsa-CircRNA_044837	hsa_circ_0044837	2.97	0.024	0.23	YPEL2	1184, 1205, 1257, 1264, 1273
					Chr.17	
hsa-CircRNA_061170	hsa_circ_0061170	2.17	0.037	0.27	RTEL1	1289, 338-3p, 370, 377, 409-3p
					Chr.20	
hsa-CircRNA_000711 *	hsa_circ_0001443	2.14	0.001	0.08	MAML3	873
					Chr.4	
hsa-CircRNA_001678 ***	hsa_circ_0000517	2.39	0.009	0.16	RPPH1	1225-3p, 1233, 1258, 1292, 1296
					Chr.14	
hsa-CircRNA_051799	hsa_circ_0051799	3.31	0.001	0.07	BAX	1827, 198, 383, 486-3p, 1180
					Chr.19	
hsa-CircRNA_000365	hsa_circ_0000365	2.07	0.005	0.13	TBCEL	1233, 1304, 339-3p, 421, 510
					Chr.11	
hsa-CircRNA_404879 ***	hsa_circ_0095784	2.22	0.026	0.24	PRR5L	1179, 1197, 1205, 1208,1229
					Chr.11	
hsa-CircRNA_000167***	hsa_circ_0000518	2.38	0.011	0.17	RPPH1	1204, 1225-3p,1233, 1258, 146b-3p
					Chr.14	
hsa-CircRNA_089763	hsa_circ_0089763	2.74	0.001	0.07	JA760600	1179, 1238, 1248, 1206, 1228
hsa-CircRNA_002082***	hsa_circ_0002082	2.45	0.045	0.29	MALAT1	1252, 217, 1208, 1224-3p, 1238
					Chr.11	
**Down-regulated CircRNAs**						
hsa-CircRNA_100332	hsa_circ_0014130	/	0.026	0.24	PIP5K1A	1205, 1246, 1182, 1183, 1184
					Chr.1	
hsa-CircRNA_004286	hsa_circ_0042860	/	<0.001	0.05	CRLF3	1827, 192, 215, 346, 516b
					Chr.17	
hsa-CircRNA_059571	hsa_circ_0059571	/	0.009	0.17	RALGAPA2	1250, 1263, 136, 1183, 1200
					Chr.20	
hsa-CircRNA_005310	hsa_circ_0005310	/	0.041	0.28	SBNO1	1246, 1251, 1264, 1290, 375
					Chr.12	

All identified circularRNAs are exonic, arising from the exons of the linear transcript, except for *: intronic, arising from an intron of the linear transcript. **: Antisense, where their gene locus overlap with the linear RNA, but transcribed from the opposite strand. ***: Sense overlapping, transcribed from same gene locus as the linear transcript, but not classified into "exonic" and "intronic". hsa: *Homo sapiens*, Circ: circular, chr: chromosome.

## Supplementary Materials

Supplementary materials can be found at http://www.mdpi.com/2073-4409/8/1/15/s1.
