# Supplementary material for: A Role for Circular Non-Coding RNAs in the Pathogenesis of Sporadic Parathyroid Adenomas and the Impact of Gender-Specific Epigenetic Regulation
Source: Cells. 2018 Dec 30;8(1):15. doi: 10.3390/cells8010015 (PMC6356744; doi:10.3390/cells8010015)
Supplement: Supplementary file 1 [file cells-08-00015-s001.zip › Supplemental Table 3_Yavropoulou.docx]

| Supplemental Table 3. Genes associated with differentially expressed circular RNAs in parathyroid adenomas from male compared to female patients | | | | |
| --- | --- | --- | --- | --- |
| Gene Symbol | **Gene Description** | **Category** | **Function** | **Gene Ontology (GO)** |
| *NEDD4L* | Neural Precursor Cell Expressed, Developmentally Down-Regulated 4-Like, E3 Ubiquitin Protein Ligase) | Protein Coding | Mediates the ubiquitination of multiple target substrates | Ubiquitin proteasome system |
| *DDX17* | DEAD-Box Helicase 17 | Protein Coding | RNA helicaseare implicated in a number of cellular processes involving alteration of RNA secondary structure, such as translation initiation, nuclear and mitochondrial splicing, and ribosome and splicesosome assembly, cellular growth and division | Nucleic acid and RNA binding |
| *DCAF17* | (DDB1 And CUL4 Associated Factor 17 | Protein Coding | A nuclear transmembrane protein that associates with cullin 4A/damaged DNA binding protein 1 ubiquitin ligase complex. | Ubiquitin proteasome system |
| *AAGAB* | Alpha And Gamma Adaptin Binding Protein | Protein Coding | The gamma-adaptin and alpha-adaptin subunits of complexes involved in clathrin-coated vesicle trafficking. | Vesicle trafficking |
| *MBNL1* | Muscleblind Like Splicing Regulator 1 | Protein Coding | A C3H-type zinc finger protein that modulates alternative splicing of pre-mRNAs | Αlternative splicing of pre-mRNAs |
| *PCBP2* | Poly(RC) Binding Protein 2 | Protein Coding |  |  |
| *MIR5695* | MIR5695( | RNA gene | Affiliated with the miRNA class. | Αlternative splicing of pre-mRNAs |
| *INCENP* | Inner Centromere Protein) | Protein Coding |  |  |
| *JA760602* | - |  |  |  |
| *YPEL2* | Yippee Like 2 | Protein Coding |  |  |
| *RTEL1* | Regulator Of Telomere Elongation Helicase 1 | Protein Coding | DNA helicase which functions in the stability, protection and elongation of telomeres and interacts with proteins in the shelterin complex known to protect telomeres during DNA replication | Nucleic acid binding and ATP-dependent helicase activity. |
| *MAML3* | Mastermind Like Transcriptional Coactivator 3 | Protein Coding | Acts as a transcriptional coactivator for NOTCH proteins. Has been shown to amplify NOTCH-induced transcription of HES1. | Transcription coactivator activity |
| *RPPH1* | Ribonuclease P RNA Component H1 | RNA Gene | H1RNA is the RNA component of the RNase P ribonucleoprotein, an endoribonuclease that cleaves tRNA precursor molecules to form the mature 5-prime termini of their tRNA sequences | Αlternative splicing of pre-mRNAs |
| *BAX* | BCL2 Associated X, Apoptosis Regulator | Protein Coding | Belongs to the BCL2 protein family. BCL2 family members form hetero- or homodimers and act as anti- or pro-apoptotic regulators that are involved in a wide variety of cellular activities. This protein forms a heterodimer with BCL2, and functions as an apoptotic activator | Protein homodimerization activity and protein heterodimerization activity. |
| *TBCEL* | Tubulin Folding Cofactor E Like | Protein Coding | Acts as a regulator of tubulin stability. |  |
| *PRR5L* | Proline Rich 5 Like | Protein Coding | Associates with the mTORC2 complex that regulates cellular processes including survival and organization of the cytoskeleton. Among its related pathways are PI3K / Akt Signaling and mTOR signalling. | Ubiquitin protein ligase binding |
| *MALAT1* | Metastasis Associated Lung Adenocarcinoma Transcript 1 | non-protein coding RNA gene | This gene produces a precursor transcript from which a long non-coding RNA is derived by RNase P cleavage of a tRNA-like small ncRNA (known as mascRNA) from its 3' end. It may act as a transcriptional regulator for numerous genes, including some genes involved in cancer metastasis and cell migration, and it is involved in cell cycle regulation. Its upregulation in multiple cancerous tissues has been associated with the proliferation and metastasis of tumor cells |  |
| *PIP5K1A* | Phosphatidylinositol-4-Phosphate 5-Kinase Type 1 Alpha | Protein Coding | Catalyzes the phosphorylation of phosphatidylinositol 4-phosphate (PtdIns4P) to form phosphatidylinositol 4,5-bisphosphate (PtdIns(4,5)P2 | Kinase binding and 1-phosphatidylinositol-4-phosphate 5-kinase activity. |
| *CRLF3* | Cytokine Receptor Like Factor 3 | Protein Coding | Negatively regulate cell cycle progression at the G0/G1 phase. Diseases associated with CRLF3 include Neurofibromatosis, Type I. | Cell cycle |
| *RALGAPA2* | Ral GTPase Activating Protein Catalytic Alpha Subunit 2 | Protein Coding | Among its related pathways are vesicle-mediated transport and translocation of GLUT4 to the plasma membrane. | Protein heterodimerization activity and GTPase activator activity. |
| *SBNO1* | Strawberry Notch Homolog 1 | Protein Coding |  | Oxidoreductase activity and hydrolase activity |
