# Supplementary material for: A Role for Circular Non-Coding RNAs in the Pathogenesis of Sporadic Parathyroid Adenomas and the Impact of Gender-Specific Epigenetic Regulation
Source: Cells. 2018 Dec 30;8(1):15. doi: 10.3390/cells8010015 (PMC6356744; doi:10.3390/cells8010015)
Supplement: Supplementary file 1 [file cells-08-00015-s001.zip › Supplemental Table 2_Yavropoulou.docx]

| Supplemental Table 1. Genes associated with differentially expressed circular RNAs in parathyroid adenomas compared to normal parathyroid tissue | | | | |
| --- | --- | --- | --- | --- |
| Gene  Symbol | **Gene Description** | **Category** | **Function** | **Gene Ontology**  **GO** |
| RPS6KA3 | Ribosomal protein S6 kinase alpha-3 | Protein Coding | Cell cycle regulation by phosphorylating the CDK inhibitor CDKN1B. | *Transferase activity, transferring phosphorus-containing groups* and *protein tyrosine kinase activity*. |
| KRT14 | Keratin 14, type I | Protein Coding | Promotes KRT5-KRT14 filaments to self-organize into large bundles and enhances the mechanical properties involved in resilience of keratin intermediate filaments in vitro. | *Structural molecule activity* and *keratin filament binding*. |
| KANSL1L | KAT8 regulatory NSL complex subunit 1-like protein | Protein Coding | **Non human** | - |
| MASTL | Microtubule Associated Serine/Threonine Kinase Like | Protein Coding | Serine/threonine kinase with a key role in M phase. Acts as a regulator of mitosis entry and maintenance. | *Transferase activity, transfer-ring phosphorus-containing groups* and *protein tyrosine kinase activity.* |
| RAB11FIP5 | Rab11 family-interacting protein 5 | Protein Coding | Protein trafficking from apical recycling endosomes to the apical plasma membrane. | *Rab GTPase bin-ding*  and *gamma-tubulin binding.* |
| TAF15 | TATA-Box binding protein-associated factor 15 | Protein Coding | RNA and ssDNA-binding protein with specific roles during transcription initiation at distinct promoters. Enters preinitiation complex together with the RNA polymerase II (Pol II). | *Nucleic acid bin-ding* and *nucleotide binding* |
| ABCC4 | ATP Binding Cassette Subfamily C Member 4 | Protein Coding | ABC proteins transport various molecules across extra- and intra-cellular membranes. This protein is a member of the MRP subfamily, which is involved in multi-drug resistance and plays a role in cellular detoxification as a pump for its substrate, organic anions. | *ATPase activity* and *15-hydro-xyprostaglandin dehydro-genase (NAD+) activity*. |
| DDX6 | DEAD-Box Helicase 6 | Protein Coding | RNA helicase, functions in translation suppression and mRNA degradation. It is required for microRNA-induced gene silencing. | *Nucleic acid binding*  and *protein domain specific binding.* |
| CTCF | CCCTC-Binding Factor | Protein Coding | Chromatin binding factor that binds to DNAActs as transcriptional repressor. Acts as tumor suppressor with a critical role in the epigenetic regulation. | *DNA binding transcription factor activity* and *chromatin binding* |
| RPPH1 | Ribonuclease P RNA Component H1 | RNA gene | **Non-Human** | - |
| OVOL2 | Ovo Like Zinc Finger 2 | Protein Coding | Zinc-finger transcription repressor factor | *Transcription regulatory region DNA binding* and *RNA polymerase II transcription factor activity, sequence-specific DNA binding*. |
| PITPNB | Phosphatidylinositol transfer protein beta | Protein Coding | Catalyzes the transfer of PtdIns and phosphatidylcholine between membranes from the Golgi to the endoplasmic reticulum. | *Lipid binding*. |
| PTN | Pleiotrophin | Protein Coding | Secreted growth factor -mitogenic for fibroblasts, epithelial, and endothelial cells | *Growth factor acti-vity* and *protein phosphatase inhibitor activity*. |
| FAM120B | Constitutive coactivator PPARG | Protein Coding | Transactivator of PPARG and ESR1. Functions in adipogenesis through PPARG activation. |  |
| ZNF652 | Zinc finger protein 652 | Protein Coding | Transcriptional repressor | *Nucleic acid binding.* |
| SUSD1 | Sushi domain-containing protein 1 | Protein Coding | Calcium ion binding-Tic Disorder | *Calcium ion binding*. |
| STIL | SCL-interrupting locus protein | Protein Coding | Plays an important role cellular growth and proliferation_decreases CDK1 activity. | - |
| LINC00969 | Long Intergenic Non-Protein Coding RNA 969 | RNA Gene |  |  |
| COL6A2 | Collagen Type VI Alpha 2 Chain | Protein Coding | Cell-binding protein. |  |
| BCAT2 | Branched-chain-amino-acid aminotransferase, mitochondrial | Protein Coding | Catalyzes the first reaction in the catabolism of the essential branched chain amino acids leucine, isoleucine, and valine. | *Branched-chain-amino-acid transaminase activity* and *L-valine transaminase activity.* |
| PIK3C2B | Phosphatidylinositol 4-phosphate 3-kinase Catalytic subunit type 2 beta | Protein Coding | Phosphorylates PtdIns and PtdIns4Pinvolved in EGF and PDGF signaling cascades. |  |
| FLNA | Filamin-A | Protein Coding | Promotes orthogonal branching of actin filaments and links actin filaments to membrane glycoproteinsAnchors various transmembrane proteins to the actin cytoskeleton and serves as a scaffold for a wide range of cytoplasmic signaling proteins. | *Transcription factor binding*. |
| THBS1 | Thrombospondin-1 | Protein Coding | Adhesive glycoprotein that mediates cell-to-cell and cell-to-matrix interactions. | *Calcium ion binding* and *heparin binding* |
| PLXNC1 | Plexin-C1 | Protein Coding | Plexins are transmembrane receptors for Semaphorins. Receptor for SEMA7A, riggers cellular responses leading to the rearrangement of the cytoskeleton and to secretion of IL6 and IL8. | *Signaling receptor binding.* |
| TROAP | Trophinin Associated Protein  (Tastin) | Protein Coding | Cell adhesion molecule complex |  |
| LTBP2 | Latent-transforming growth factor beta-binding protein 2 | Protein Coding | Member of the TGF-beta latent complex, function as a structural component of microfibrils, with a role in cell adhesion. | *Calcium ion bind-ing* and *growth factor binding*. |
| FN1 | Fibronectin 1 | Protein Coding | Cell adhesion, cell motility, opsonization, wound healing, and maintenance of cell shape. | *Heparin binding* and *protease binding*. |
| CD74 | HLA class II histocompatibility antigen gamma chain | Protein Coding | Associates with class II major histocompatibility complex (MHC) and is an important chaperone that regulates antigen presentation for immune response. | *Identical protein binding* and *amyloid-beta binding*. |
| POLR2A | DNA-directed RNA polymerase II subunit A | Protein Coding | Catalyzes the transcription of DNA into RNA . | *DNA-directed 5-3 RNA polymerase activity*. |
| CD44 | CD44 antigen | Protein Coding | Cell-surface glycoprotein involved in cell-cell interactions, cell adhesion and migration.  Receptor for hyaluronic acid (HA). | *Transmembrane signaling receptor activity* and *cytokine receptor activity*. |
| GPRC5A | Retinoic acid-induced protein 3 | Protein Coding | Orphan receptor. Functions as a negative modulator of EGFR signaling | *G-protein coupled receptor activity*. |
| PDIA4 | Protein disulfide-isomerase A4 | Protein Coding | Deoxycytidine kinase. Catalyzes the rearrangement of -S-S- bonds in proteins. | *Isomerase activity.* |
| RPL17 | Ribosomal protein L17 | Protein Coding | Component of the large ribosomal subunit 60S. | *Structural constituent of ribosome*. |
