# Supplementary material for: A Role for Circular Non-Coding RNAs in the Pathogenesis of Sporadic Parathyroid Adenomas and the Impact of Gender-Specific Epigenetic Regulation
Source: Cells. 2018 Dec 30;8(1):15. doi: 10.3390/cells8010015 (PMC6356744; doi:10.3390/cells8010015)
Supplement: Supplementary file 1 [file cells-08-00015-s001.zip › Supplemental Table 1_Yavropoulou.docx]

| Supplemental table 1. Immunohistochemical analysis of all samples included in the analysis. | | | | | |
| --- | --- | --- | --- | --- | --- |
| Sample ID | **Gender** | **APC %** | **Parafibromin expression** | **Cyclin D1 (%)** | **Ki67 (%)** |
| Sporadic Parathyroid adenomas | | | | | |
| Di_M_27 | M | 60 | + | 70 | 1 |
| Di_M_28 | M | 30 | + | 50 | 0 |
| Di_M_32 | M | 80 | + | 60 | 1 |
| Di_M_35 | M | 25 | + | 70 | 1 |
| Di_M_36 | M | 100 | + | 90 | 1 |
| Di_M_37 | M | 20 | + | 2 | 1 |
| Di_M_38 | M | 15 | + | 35 | 0 |
| Di_F_9 | F | 20 | + | 30 | 1 |
| Di_F_10 | F | 55 | + | 30 | 0 |
| Di_F_11 | F | 30 | + | 10 | 1 |
| Di_F_12 | F | 20 | + | 30 | 1 |
| Di_F_14 | F | 20 | + | 15 | 1 |
| Di_F_15 | F | 30 | + | 20 | 0 |
| Di_F_20 | F | 15 | + | 5 | 1 |
| Di_F_21 | F | 0 | + | 50 | 1 |
| Di_F_34 | M | 50 | + | 10 | 0 |
| Normal Parathyroid tissue | | | | | |
| Con_1 | F |  | + | 3 | 0 |
| Con_29 | F |  | + | 20 | 0 |
| Con_30 | F |  | + | 5 | 0 |
| Con_31 | F |  | + | 5 | 0 |

Parafibromin is evaluated as positive (+) for any percentage of nuclear positivity and negative (-) for no nuclear positivity. For APC cytoplasmic positivity was evaluated, as a percentage of positive cells. For CyclinD1 and Ki67 nuclear positivity was evaluated as a percentage of positive cells. Each stain was evaluated in three cores and the average value was taken into account for statistical analysis.

APC, of Adenomatous Polyposis Coli; M, male; F, female
