# Supplementary figures and images for: A Role for Circular Non-Coding RNAs in the Pathogenesis of Sporadic Parathyroid Adenomas and the Impact of Gender-Specific Epigenetic Regulation
Source: Cells. 2018 Dec 30;8(1):15. doi: 10.3390/cells8010015 (PMC6356744; doi:10.3390/cells8010015)

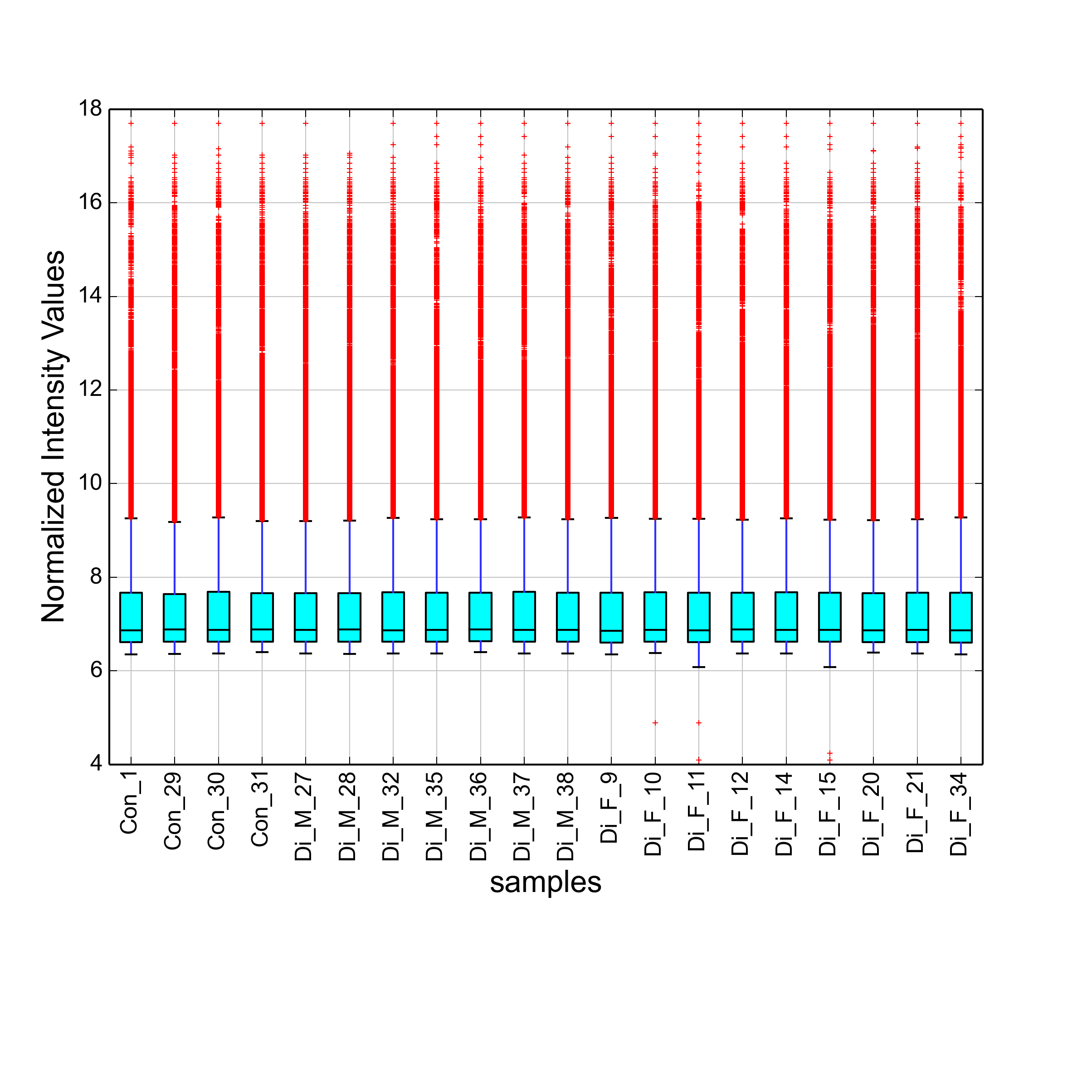


Suppl. Figure 1

**Tested Samples**

**Normal parathyroid tissue**

**Parathyroid Adenomas**

Supplement: Supplementary file 1 [file cells-08-00015-s001.zip › Supplemental Figure 1_Yavropoulou.docx]
